# Quantitative Super‐Resolution Imaging of Molecular Tension

**DOI:** 10.1002/advs.202408280

**Published:** 2025-04-17

**Authors:** Seong Ho Kim, Adam B. Yasunaga, Hongyuan Zhang, Kevin D. Whitley, Isaac T. S. Li

**Affiliations:** ^1^ Department of Chemistry University of British Columbia Kelowna BC V1V 1V7 Canada; ^2^ Department of Chemistry and Advanced Materials College of Natural Sciences Gangneung-Wonju National University Gangneung 25457 Republic of Korea; ^3^ Centre for Bacterial Cell Biology Biosciences Institute Newcastle University Newcastle upon Tyne NE1 7RU UK

**Keywords:** DNA‐PAINT, functional super‐resolution imaging, molecular beacon, molecular tension sensor

## Abstract

DNA‐based molecular tension probes have revolutionized the localization of mechanical events in live cells with super‐resolution. However, imaging the magnitude of these forces at super‐resolution has been challenging. Here, qtPAINT (quantitative tension points accumulation for imaging in nanoscale topography) is introduced as a strategy to image the magnitude of molecular tension with super‐resolution accuracy. By leveraging the force‐dependent dissociation kinetics of short DNA oligonucleotides on their complementary strands, tension is encoded on individual molecules through their binding kinetics. This method allowed for a quantitative analysis of these kinetics, providing a detailed reconstruction of the force magnitudes acting on each tension probe. The technique integrates a molecular‐beacon PAINT imager with a hairpin molecular tension probe, achieving a force quantification range of 9–30 pN and maintaining a spatial resolution of 30–120 nm in low and high‐density regions. Additionally, qtPAINT offers a temporal resolution on the order of a minute, enhancing its applicability for studying dynamic cellular processes.

## Graphical abstract

Quantitative tension PAINT (qtPAINT) is a new method to quantify molecular tension at super‐resolution by exploiting force‐dependent DNA oligonucleotide dissociation kinetics. Combining hairpin tension probes and molecular‐beacon imagers, qtPAINT achieves 9–30 pN continuous force quantification range with 30–120 nm spatial and minute‐scale temporal resolutions, enabling detailed visualization of dynamic molecular forces in focal adhesions during cellular adhesion and migration.

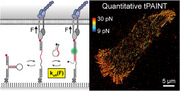

## 1 Introduction

Mechanical forces at the molecular scale play a pivotal role in the functioning of multicellular organisms. They drive critical processes, including development, tissue homeostasis, and immune responses.^[^ ^1^, ^2^, ^3^ ^]^ Within biological systems, mechanical forces in environments like tumor tissues and the bloodstream intricately govern pathways related to tumorigenesis and endothelial mechanotransduction.^[^ ^4^, ^5^ ^]^ The dynamics of mechanical forces on adhesion receptors, such as integrins, PECAM‐1 (platelet endothelial cell adhesion molecule), E‐cadherin, Talin‐1, and Notch, are crucial for mechanosensing and mechanotransduction.^[^ ^6^, ^7^, ^8^, ^9^ ^]^ The activation of these proteins is tightly linked to the intensity and duration of the forces applied. For example, a brief application of high force induces significant conformational changes in Talin‐1, exposing binding sites, whereas a similar outcome under low force requires longer exposure.^[^ ^9^ ^]^ PECAM‐1, under varying force, triggers specific downstream signaling responses on a minute scale.^[^ ^7^ ^]^ E‐cadherin modulates cell‐cell interaction‐mediated signaling in response to its adhesion strength. Therefore, discerning the spatial, temporal, and magnitude dynamics of mechanical force at the molecular level is crucial for understanding mechanosensitivity and its integration with cellular biochemical responses.

Integrin‐mediated mechanotransduction, a key mechanically‐driven cellular process, enables communication between cells and the extracellular matrix (ECM) within their microenvironment.^[^ ^10^, ^11^ ^]^ This interaction is facilitated through focal adhesions (FAs), complex assemblies of integrin clusters, signaling proteins, and regulatory factors.^[^ ^12^ ^]^ FAs evolve from integrin engagement, developing through nascent adhesions (NAs) that are essential for cell migration and the initiation of mechanotransduction^[^ ^13^, ^14^, ^15^ ^]^ and develop through focal complexes to mature FAs.^[^ ^16^, ^17^, ^18^ ^]^ Despite their significance, the dynamics of molecular forces involved in these processes remain elusive, constrained by the limited capabilities of current techniques to map the magnitudes of these forces with high spatial and temporal resolutions. This knowledge gap significantly impedes our ability to fully decipher the complex relationship between mechanical forces at the molecular level and the subsequent cellular responses they elicit. Therefore, it is vital to have techniques that can resolve the magnitude of molecular forces with high spatial and temporal resolution to understand how their dynamics regulate cellular functions.

Various methodologies have been employed to unravel cellular force dynamics at macroscopic and molecular levels.^[^ ^19^, ^20^ ^]^ Traction force microscopy, a macroscopic technique, has been extensively utilized to study cellular tension. It offers exceptional whole‐cell level force readouts and high temporal resolution. However, it cannot resolve molecular and nanoscale forces, particularly those generated by Nas and individual adhesion events, due to resolution constraints.^[^ ^21^, ^22^, ^23^ ^]^ At the molecular scale, the development of molecular tension probes over the past decade has been significant, with various reviews offering perspectives on their design, classification, and interpretation.^[^ ^20^, ^24^, ^25^ ^]^ Elastomeric peptide‐based probes, for instance, utilize Förster Resonance Energy Transfer (FRET) to deduce the force magnitude on individual sensors.^[^ ^26^, ^27^, ^28^ ^]^ However, the FRET signal represents an average of all probes within the optical resolution limit, and the properties of the elastomeric peptide constrain its dynamic range. DNA‐based probes have also been instrumental in studying molecular forces. Unlike elastomeric peptides, these probes detect mechanical events through reversible two‐state conformational changes (e.g., hairpin probes^[^ ^29^, ^30^ ^]^) or irreversible dissociation (e.g., TGTs – tension gauge tethers^[^ ^31^ ^]^). The resultant fluorescence signal is binary, reflecting a threshold level (i.e., F_½_ for hairpin probes or a loading‐rate‐dependent rupture force in the case of TGTs).^[^ ^29^, ^30^, ^32^, ^33^ ^]^ Consequently, the total fluorescence signal corresponds directly to the number of activated sensors but does not explicitly reveal the force magnitude. The recent advent of super‐resolution tension imaging, combining DNA hairpin probes and PAINT, has provided exceptional spatial resolution in visualizing mechanical events.^[^ ^30^, ^33^ ^]^ We have further advanced this field by incorporating molecular beacons (MBs) into tension PAINT imaging, significantly enhancing fluorescence background reduction and imaging speed and improving the signal‐to‐noise ratio for live‐cell tension imaging.^[^ ^34^ ^]^ Despite these advancements, achieving super‐resolution molecular tension imaging with quantifiable force magnitude and capturing the force dynamics over time remains challenging. Current tension PAINT methods provide only a binary readout: 1) force is detected only if it exceeds the threshold to unfold the probe (e.g., DNA hairpin) and 2) once unfolded, PAINT imager binding produces discrete “on” signals. As a result, existing approaches reveal the location of mechanical events but not their actual force magnitudes.

To address this limitation, we introduce a quantitative method for super‐resolution tension PAINT (points accumulation for imaging in nanoscale topography) that leverages the force‐dependent dissociation kinetics of DNA oligonucleotides under mechanical tension. This approach not only localizes mechanical events but also recovers the magnitude of the forces involved. By correlating the tension experienced by individual molecules with these dissociation kinetics, we establish a direct link between kinetic behavior and mechanical forces. Employing single‐molecule kinetics analysis of these binding events allows us to reconstruct the mechanical force exerted on each molecular tension probe. Our method achieves a dynamic force quantification range of 9–30 piconewtons with a spatial resolution of 30–120 nm and a temporal resolution on the order of minutes. This capability enables a detailed and dynamic characterization of molecular forces fundamental to cellular mechanics. Our findings illuminate the regulation of cell adhesion across a spectrum of forces, and the mean tension distribution offers new insights into the relationship between local, dynamic tensions and cellular functions in adhesion and motility.

## 2 Results

### 2.1 Principle of Kinetic‐Based Molecular Tension Quantification

Previous studies using optical tweezers have demonstrated a significant force dependency in the binding kinetics of short oligonucleotides (8–12 nucleotides) to their complementary strands under tension, covering a wide force range.^[^ ^35^ ^]^ Notably, the dissociation rate (*k_off_*) escalated substantially with increasing force, showing nearly a tenfold increase for every 10 pN of force applied. Conversely, the association rate was less affected by force, with changes confined to within a four‐fold across a 0–50 pN force range. Building upon these findings, we integrated a force‐dependent kinetics model with an MB imager^[^ ^34^ ^]^ and hairpin tension probes to establish a quantitative tension PAINT (qtPAINT) imaging technique. This approach enables us to determine the magnitude of force on individual tension probes with super‐resolution precision.

**Figure 1:**
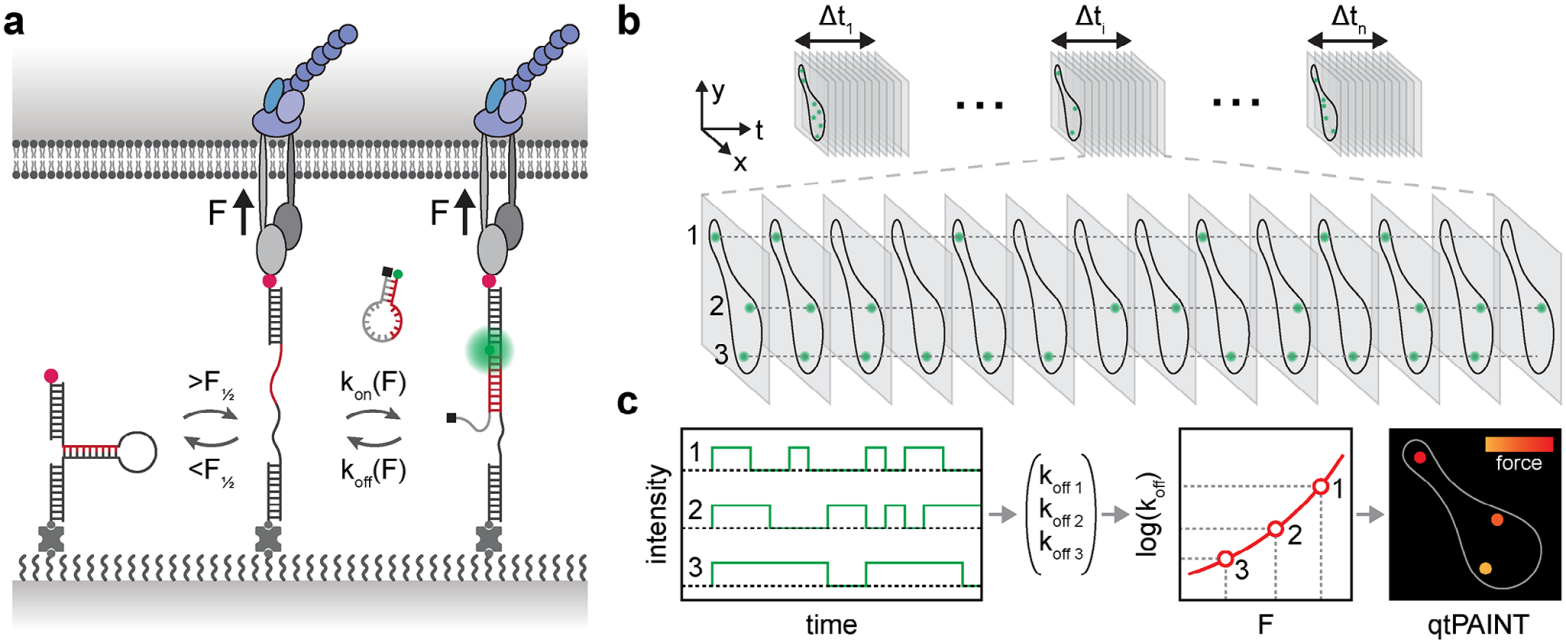
Schematic of quantitative tension PAINT (qtPAINT) imaging. a) Probe design and mechanism. The qtPAINT technique utilizes a DNA hairpin tension sensor marked with a cRGDfk peptide (red dot). This sensor can reversibly open when the integrin‐mediated tension (*F*) surpasses the threshold (*F_½_*), revealing a target binding site (red line). This site experiences the same *F*. The binding kinetics (*k_on_* and *k_off_*) of the MB to this exposed site are governed by the magnitude of integrin tension. b) The PAINT image can be divided into segments of arbitrary duration of *Δt_i_*, depending on the desired temporal resolution. Localized tension events are identified and connected within each segment by single‐molecule tracking. c) Kinetic analysis and tension mapping. A kinetic profile is established for each molecular binding event based on single‐molecule tracking data. *k_off_* is calculated from these kinetic profiles for each track. These *k_off_* values are converted into force measurements using a force‐dependent kinetic model.^[^ ^35^ ^]^ Calculated force values are color‐coded to generate a detailed functional tension map representing both the distribution and the magnitude of molecular forces for each *Δt*. Representative qtPAINT traces and subsequent analysis and mapping are shown in Figure S6 (Supporting Information).

We designed reversible hairpin tension probes incorporating an opening force (F_½_) of ≈9 pN based on our previous construct (Table S1, Supporting Information).^[^ ^29^, ^34^ ^]^ These probes are engineered with biotin at one end for surface attachment and a cRGDfk peptide at the other for integrin binding (**Figure** **1**a). Upon application of tension exceeding F_½_, the hairpin structure unfolds, exposing a previously concealed cryptic binding site for the MB imager. Our MB imager is designed with a short stem and a large loop to ensure fast binding kinetics and effective quenching before target binding. The binding of the MB with the cryptic site triggers the separation of the stem, resulting in the dequenching of the Cy3B fluorophore and producing a detectable localization signal. The association (*k_on_*) and dissociation (*k_off_*) rates of this interaction are force‐dependent, with *k_off_* being directly observable and quantifiable through the duration of the binding events. As demonstrated in our earlier work, fluorogenic MB imagers significantly reduce background interference, allowing for a higher imager concentration. This enhances localization event density without adversely affecting background fluorescence, improving overall imaging speed. This advancement is crucial since the tension applied shortens the binding lifespan of the MB to the hairpin tension probe, necessitating faster imaging speeds.

The PAINT movie can also be segmented into time bins of arbitrary lengths (*Δt_i_*) to capture temporal dynamics of force (Figure 1b). We employed single‐molecule tracking to create the blinking trajectories of individual adhesion events. This allows us to extract kinetic data of forces, particularly *k_off_*, at each hairpin tension probe (Figure 1c). By applying the established force‐dependent kinetic model,^[^ ^35^ ^]^ we could infer the tension (*F*) from *k_off_* values. When combined with spatial localization, this process (Figure 1c) generates a functional super‐resolution tension map. Iterating this procedure across successive segments allows us to construct a detailed spatio‐temporal map with the magnitude of forces, enabling the investigation of the dynamics of molecular forces involved.

### 2.2 Quantitative Tension Imaging Unveils the Structure, Dynamics, and Force Magnitude of Adhesion Events

**Figure 2:**
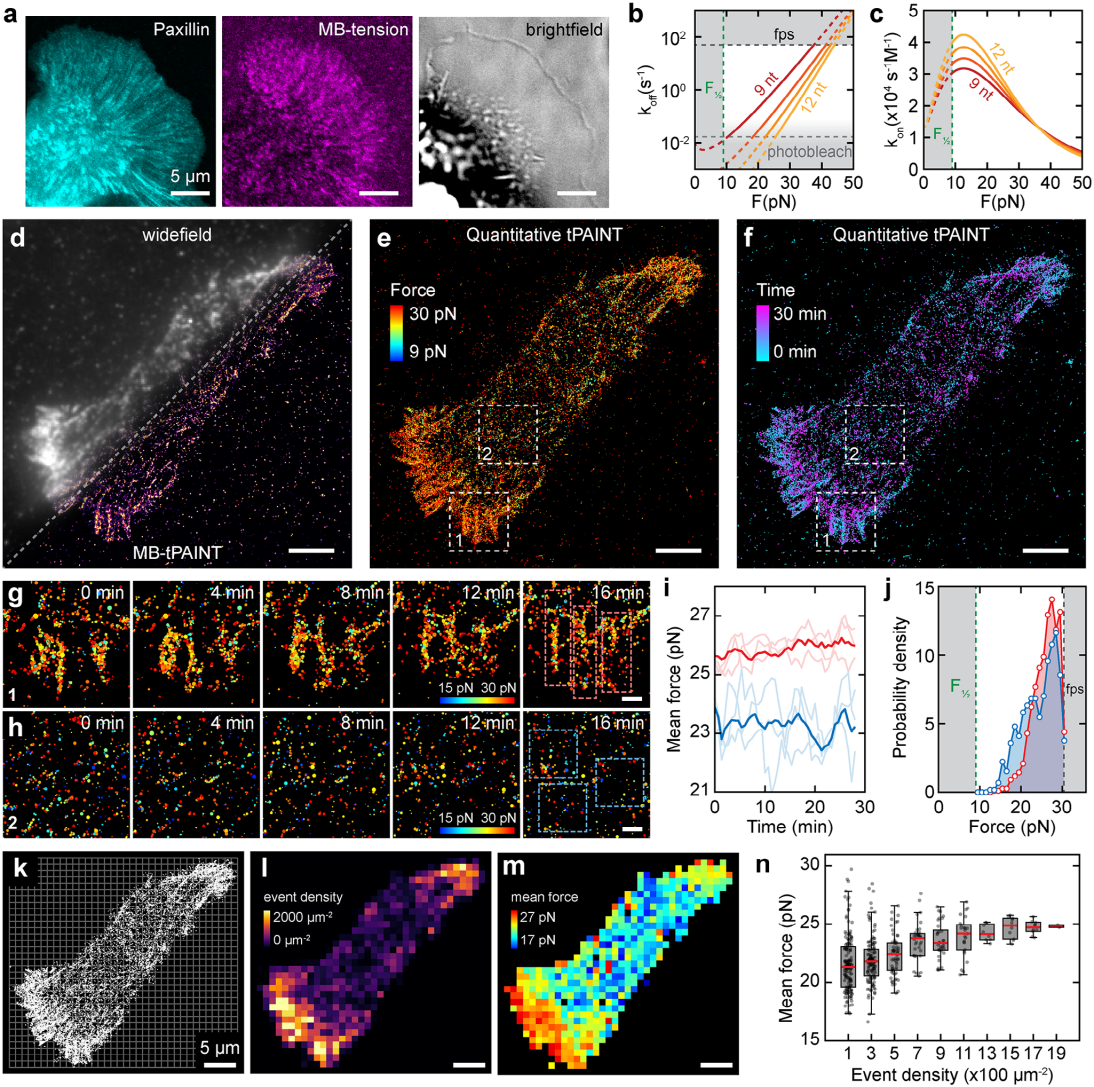
Live cell analysis using quantitative tension PAINT imaging. a) Confocal images of Paxillin (cyan), tension (purple), and brightfield (gray) of a CHO‐K1 cell overexpressing GFP‐Paxillin. b,c) The kinetic model illustrates the force‐dependent k_off_ and k_on_ modeled for MB hybridization lengths from 9 nt (red) to 12 nt (yellow). The frame per second (fps) rate limits the upper force detection range (50 fps shown). The hairpin‐opening force *F_1/2_* and the photobleaching rate constrain the lower force detection range. d) Widefield and super‐resolved tension image using MB‐based tension PAINT. e) Quantitative tension map with forces color‐coded between 9–30 pN. f) Temporal dynamic map with the onset time of individual force events color‐coded from 0–30 mins. g,h) Time series of zoomed‐in ROI1 and ROI2 from e,f), captured at 4‐min intervals. i) Mean force trajectories (light red and blue) of three individual elongated FAs from the ROIs in g) and three random ROIs from the cell center h), along with their averaged trajectories of elongated FAs (solid red) and cell interior (solid blue). j) The probability density distribution of molecular force in ROI1 (red) and 2 (blue). Shaded areas represent regions where force quantification is unattainable due to the opening force of the hairpin probe and the acquisition frame rate (5 fps). k–m) Coarse‐grained grid maps showing the grid layout (k), event density (l), and mean force (m) with a grid size of 1 × 1 µm. n) Box plot of mean force versus event density with each data point representing values from a single grid in (k‐m). Event density binned at 200 µm^−2^ per bin. Scale bars: 5 µm (a, d–f, k–m) and 1 µm (g, h).

First, we confirmed that cells form robust adhesive structures on surfaces coated with hairpin tension probes, and these tension events are effectively visualized using our MB imagers. The engagement of external integrin with the substrate initiates cytoskeleton assembly, signaling proteins, and integrin clustering. This results in the formation of mature FAs, typically characterized by elongated morphologies extending over several micrometers.^[^ ^36^, ^37^ ^]^ Cells anchored to DNA sensor‐modified surfaces exhibited tension signals, appearing as intense, concentrated puncta predominantly around the cell peripheries (**Figure** **2**a). We observed that these signals colocalize with FA proteins such as Paxillin and Talin‐1, forming rod‐shaped FAs, confirming the origination of tension within these structures (Figure 2a; Figures S1,S2, Supporting Information). The distribution of Paxillin in punctate formations along polymerizing actin filaments, coupled with their retrograde movement driven by myosin contraction, reflects the directional nature of cellular tension (Movies S1 and S2, Supporting Information). Additional control (Figure S3, Supporting Information) showed that attaching the integrin ligand (cyclic RGD) to the hairpin probe is required to produce a fluorescence signal with MB‐PAINT imagers. Together, the above evidence indicates that the fluorescence signals observed directly result from tension through integrin engagement.

We modeled the force‐dependent DNA hybridization kinetics of our MB imagers with various binding sequence lengths (9 to 12 nucleotides) using an experimentally validated theoretical model (Figure 2b).^[^ ^35^ ^]^ We observed that the dissociation rate constant (*k_off_*) increased monotonically with force, starting from a few pNs. Theoretically, this suggests a broad force quantification range, from a few piconewtons to potentially infinite values. However, our experimental detection range is constrained by three technical factors. First, the lower limit of force detection is set by the F_1/2_ of the hairpin probe (9 pN in our case), which is essential for discriminating force‐activated signals from non‐tensioned ones. Additionally, the bound lifetime of MB imagers at low forces would reach the same time scale of photobleaching (Figure S15, Supporting Information), thus setting another lower boundary for force detection. Although this is not a sharp boundary, the inability to distinguish photobleaching events from unbinding events lowers the quantification confidence if the binding lifetime scale approaches photobleaching. Finally, the acquisition frame rate determines the upper limit of force quantification, as we cannot measure *k_off_* faster than this rate. Consequently, these constraints set the force quantification range for the 9 nt MB imager at ≈9–37 pN and the 12 nt MB imager at ≈25–44 pN (Figure 2b) at 50 frames per second (fps) acquisition rate.

It is important to note that while *k_off_* increases exponentially with force, the association rate constant (*k_on_*) exhibits a weaker force dependency. Over the force range of 9–50 pN, *k_on_* changes by a maximum of eight‐fold and only about two‐fold within our *k_off_*‐limited force detection range as predicted by the model. However, *k_on_* is not a reliable parameter for force quantification for several reasons: 1) *k_on_* values are not strictly one‐to‐one functions of force at lower force ranges, 2) accurate force determination requires knowledge of the exact local imager concentration, 3) the binding kinetics of MB imagers are roughly 70 times slower than linear imagers due to the additional step of stem opening before target binding,^[^ ^34^ ^]^ which requires more detailed characterization before a quantification effort, and 4) the relatively minor variations in *k_on_* across the force range necessitate a significantly higher number of localization events for force differentiation statistically. Therefore, in this study, we exclusively utilize *k_off_* for force quantification.

To observe adhesion structures, we conducted tension PAINT experiments using MB imagers on a polarized cell (Figure 2d). In live cell tension imaging, the spatial resolution is influenced by integrin density and tension levels. In mature FAs where integrin cluster density exceeds the spatial resolution, kinetic events from different probes cannot be easily separated, reducing the spatial resolution of force. Consequently, the spatial resolution of force is not necessarily the spatial resolution of localization and varies across regions with different integrin densities. We observed the force spatial resolutions of 120 nm (4σ) in mature FAs with high integrin density and ≈30 nm (localization precision, σ) in low integrin density regions outside of FAs (Figure S4b, Supporting Information). We identified elongated FAs predominantly at polarized edges of the cell, indicative of mature FAs, in contrast to smaller adhesion clusters or events (<200 nm) observed in the cell center. Utilizing the kinetic model (Figure 2b) and our analysis pipeline (Figure 1b,c), we transformed blinking kinetics into force data (see sample traces in Figure S6, Supporting Information), generating a super‐resolved force map that displayed the magnitude of individual adhesion events in a living cell (Figure 2e).

Moreover, the onset frame of each mechanical event provides the temporal dynamics of force (Figure 2f), allowing us to image mechanical events at super‐resolution with both magnitude and time information, providing details in the spatio‐temporal‐magnitude dynamics of molecular adhesion. The force magnitude map showed that higher tensions were primarily localized at the polarized cell edges, where retraction occurred, while weaker tensions were observed in the perinuclear and nuclear regions, where the cell remained stationary (Figure 2e). The temporal map depicted an orchestrated movement of FAs, initiating at the periphery and contracting toward the center over a 30‐min period.

Next, we closely examined two regions of interest (ROIs, Figure 2e) to compare the molecular force dynamics at polarized edges (with mature FAs, Figure 2g) and the cell interior (isolated mechanical events, Figure 2h) with detailed statistics of kinetic parameters shown in Figure S7 (Supporting Information). The three closely situated FAs (Figure 2g) displayed a similar trend of increasing mean molecular force with time as the cell contracted (Figure 2i). In contrast, adhesion events in the cell interior (Figure 2h) demonstrated significantly more dynamics, characterized by a lack of structured formations, greater force fluctuations, and a lower mean force (Figure 2i). This observation was further corroborated when we examined the molecular force distribution between these two areas, revealing a significantly larger population of molecular forces in the 10–22 pN range in the interior (Figure 2j).

While qtPAINT reconstructions (Figure 2g,h) provide excellent resolution and the most information, the large tension diversity (Figure 2j–n) makes it hard to visualize patterns. To better visualize molecular force over the entire cell, we constructed a coarse‐grained grid map (Figure 2k). In this map, we quantified the density of molecular tension events and the mean force within each grid (Figure 2l,m). We noted a higher density of tension events at the polarized edges of the cell, which decreased significantly toward the center (Figure 2l). Similarly, the mean force at the polarized edges reached ≈27 pN, compared to ≈17 pN in the cell interior (Figure 2m). These force magnitudes are consistent with previously estimated molecular force ranges for CHO‐K1 cells using TGTs.^[^ ^38^ ^]^

By analyzing the relationship between mean force and event density, we observed a general trend: regions with higher tension density exhibited increased mean force and reduced force variability (Figure 2n). Notably, the significant force heterogeneity in areas with low event density, primarily the cell's interior, is likely due to greater force fluctuations (Figure 2i) and fewer events available for statistical averaging. To further understand subcellular structural mechanics, we segmented a polarized cell into three sub‐areas along the center and edges (Figure S5a–c, Supporting Information). The central areas consistently showed lower tensions than the edges (Figure S5b–e, Supporting Information). Additionally, adjacent focal adhesion areas at the bottom edge (E1, E2) exhibited tension dynamics distinct from those on the opposite side of the cell (E3). A mature FA‐masked image (Figure S5d, Supporting Information) revealed isolated tension islands at the top and bottom edges along the polarization axis. Although tensions across different regions were statistically distinct, their magnitudes were overall similar (Figure S5d,e, Supporting Information).

Previous studies have reported tensions over 7 pN across talin‐1 and only 2.5 pN across vinculin in FAs.^[^ ^26^, ^27^ ^]^ There is also an intramolecular tension gradient across talin during integrin‐mediated cell adhesion, highlighting varied tensions across the talin head domain, the rod domain, and among actin‐binding sites within the rod domain.^[^ ^27^ ^]^ The integrin tension measured using qtPAINT ranged from 10–30 pN and beyond (Figure 2j–n), surpassing the tension across individual talin‐1 and vinculin molecules. This suggests that the tension transmitted through the focal adhesion complex in individual integrin adhesions is distributed across various components, reflecting the combined effects of multiple proteins such as actin, myosin, talin‐1, and vinculin, contributing to the broad range of integrin tensions observed.

### 2.3 Molecular Adhesion Force Dynamics in Stationary and Migratory Cells Using qtPAINT

**Figure 3:**
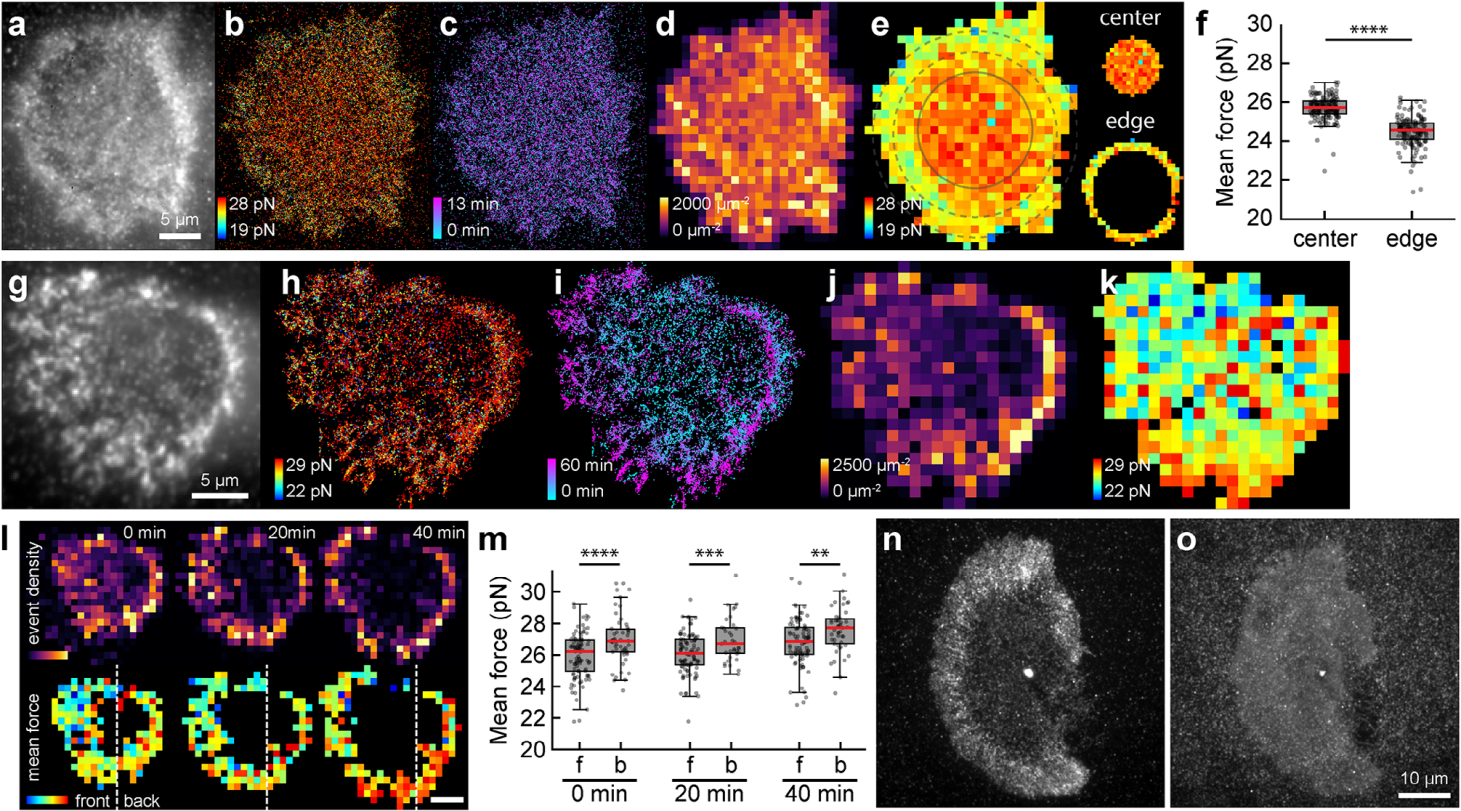
Molecular tension distribution in stationary unpolarized and directionally migrating cells using qtPAINT. a–e) Unpolarized Stationary CHO‐K1 cell tension imaging and analysis, showing diffraction‐limited tension image (a), super‐resolved tension magnitude (b) and temporal (c) images, cumulative adhesion event density map (d) and mean force map (e) over a 13‐min period. The central (within solid line) and peripheral (between dashed lines) regions of the stationary cell were analyzed to compare mean force values in f). g–i) Tension imaging of a directional migrating CHO‐K1 cell in a similar set of images showing diffraction‐limited tension image (g), super‐resolved tension magnitude (h) and temporal (i) images, cumulative adhesion event density map j) and mean force map k) over a 60‐min period. l) Adhesion density and mean force in 20‐min segments. A White dashed line marks the cell's leading (front or “f”) and trailing (back or “b”) edges. m) Quantification of average force at the leading and trailing edge of the cell in (l), showing statistically greater force at the trailing edge throughout the cell's directed migration. (n, o) Diffraction‐limited tension images of a HEK293 cell before n) and 20 mins after o) 25 µm blebbistatin treatment. Scale bars: 5 µm (a–i) and 10 µm (n,o).

We first analyzed the molecular adhesion force distribution and dynamics in stationary cells with an unpolarized morphology. These cells presented a circular shape and uniformly distributed adhesion events across the cell area, with no discernible FAs. During a 13‐min observation, these adhesion events appeared and disappeared stochastically, showing no apparent patterns or coordinated movements (**Figure** **3**a–c), indicating a lack of dynamic FA protein assembly and growth. Despite the uniform presence of fluctuating adhesion events, the molecular force distribution was uneven (Figure 3d,e). The outer regions of the cell consistently generated lower tension than the central regions (Figure S8, Supporting Information). The force dynamics exhibited similar patterns across different areas in this morphological and adhesion state (Figure S8, Supporting Information). We noted a statistically significant increase in mean force — by ≈1.2 pN — from the cell periphery toward the center (Figure 3f), a trend not associated with the density of adhesion events. This pattern contrasted with that in polarized cells, where mature FAs exhibited higher mechanical event density and greater force magnitude (Figure 2l–n). Interestingly, this central tension, greater than peripheral tension, was consistent across various cell lines, including HEK293 and MDA‐MB‐231 (Figures S9,S10, Supporting Information). In MDA‐MB‐231 cells, we noted a unique ring‐structured adhesion event at the periphery, which, despite its high mechanical event density, exhibited lower tension than the cell interior (Figure S10, Supporting Information), a phenomenon also observed in platelet cells.^[^ ^33^ ^]^ To further explore this phenomenon, we analyzed and compared tension distributions in stationary, unpolarized cells between the HEK293 and CHO‐K1 cell lines. Both cell lines consistently exhibited higher tensions in the central regions than the edges (Figure S8b–d, Supporting Information), with similar overall tension distributions across multiple cells of both lines (Figure S11c,e,f, Supporting Information). This suggests that integrin tension and function may be conserved across species, requiring similar molecular tension even in diverse biological contexts.

Next, we shift our analysis to motile cells exhibiting directional movement. These cells showed tensional polarization at both the leading and trailing edges, with dynamic tension observed during migration and spreading (Figure 3g–m). This directional migration is driven by cytoskeletal reorganization and actomyosin contractility, with actin assembly at the leading edge forming lamellipodia and disassembly at the trailing edge, facilitating detachment and retraction.^[^ ^39^, ^40^, ^41^ ^]^ Over 60 min, we imaged migrating cells to produce super‐resolved images of tension dynamics and grid maps of event density and tension (Figure 3h–k). The cell's movement was evidenced by the color gradient in the time‐colored image (Figure 3i). Unlike stationary cells, migratory cells concentrate most tension events and their magnitudes along the cell edges. To analyze tension differences between the leading and trailing edges, we divided the image into halves, designating the left as the leading edge and the right as the trailing edge (Figure 3l). Initially, the leading edge showed 2.5% lower mean tension than the trailing edge, but this difference expanded to 3.2% as cell movement and spreading intensified (Figure 3m). This pattern of higher tension at the trailing edge, attributed to contractile forces in the actomyosin network, and dynamic, heterogeneous tension at the leading edge, is consistent with existing literature.^[^ ^39^, ^42^, ^43^ ^]^ Treatment with blebbistatin, a myosin II inhibitor, led to a loss of tension signal after 13 min (Figure 3n,o), underscoring the role of actomyosin contractility in generating these signals. Monitoring post‐treatment showed a significant decrease in tension magnitude within 200 s (Figure S12, Supporting Information), further validating that qtPAINT force quantification accurately reflects cellular mechanical processes. Lastly, our control experiment of fixed cells demonstrated minimal changes in kinetics over time (Figure S13, Supporting Information), further demonstrating that time‐dependent kinetic changes result from tension.

## 3 Discussion

The force‐dependent *k_off_* model was developed based on single‐molecule optical tweezer studies.^[^ ^35^ ^]^ While the effects of salt in cell media can be accounted for using DNA hybridization models, the influence of complex extracellular factors such as molecular crowding, charge interactions, and confinement due to proximity to cell surface lipids, proteins, and glycans is less understood. Therefore, although in vitro kinetic characterization provides a robust estimate of forces, absolute force quantification remains challenging due to the complexity of the cellular environment. It is nearly impossible to account for every potential factor affecting force measurements at the single‐molecule level, and thus, our force values are derived from in vitro experimental models. The approach of applying in vitro characterizations to in vivo force probes is well established in molecular mechanotransduction studies.^[^ ^29^, ^31^ ^]^ Furthermore, our force measurements align with previous estimates using vinculin and talin tension sensors.^[^ ^26^, ^27^ ^]^ In qtPAINT, while such variables may influence imager diffusion and its *k_on_*, they are less likely to impact *k_off_(F)*, which is primarily governed by mechanical force. Understanding how the cellular environment alters the behavior of molecular force probes would require further experimental investigation for this field to move forward.

Minimizing non‐specific localization signals is critical in qtPAINT, as false signals can be misinterpreted as mechanical events and bias force quantification. In particular, non‐specific localization events are often short‐lived and indistinguishable from high‐force events. Since force determination is based on unbinding kinetics, *k_off_*‐based filtering cannot be applied, making it essential to suppress non‐specific events. Fluorogenic MB imagers effectively mitigate this issue by producing localization signals only upon target binding, minimizing artifacts from non‐specific surface binding.^[^ ^34^ ^]^ Additionally, the lower *k_on_* of MB imagers reduces the likelihood that the same MB imager binds immediately to an adjacent target upon dissociating from its current target, reducing the probability of combining distinct events in proximity as a single binding event, thereby enhancing the accuracy of *k_off_* assessment. The force quantification can be further improved by excluding low‐brightness and low‐event traces (Figure S7, Supporting Information) to minimize localization artifacts and low‐confidence quantifications.

Expanding the *k_off_* detection range is important for broadening the force quantification range, as every 10 pN increment in force corresponds to roughly one order of magnitude change in *k_off_*. However, this expansion is constrained by factors such as the binding sequence length, imaging speed, and phototoxicity. For instance, a 12 nt imager theoretically extends force quantification up to ≈50 pN at 1000 fps with the latest sCMOS cameras (Figure 2b). However, increasing the binding sequence length raises the lower force detection limit, as *k_off_* at low forces becomes so slow that photobleaching dominates the bound‐state lifetime, limiting the lower force quantification bound.

Phototoxicity presents another challenge for long‐term, high‐resolution force imaging. Reducing cell death during extended observations (up to 60 min) required us to use a low laser power setting (2–5 mW, Table S2, Supporting Information). This reduced both phototoxicity and fluorophore photobleaching, which improves the low‐force quantification range. However, this requires a trade‐off between spatial resolution and frame rate, affecting the upper force quantification limit. Our current approach achieved 30–120 nm spatial resolution at 5 fps using a Cy3B fluorophore and 2 mW laser power (Figure 2b). This enabled us to quantify *k_off_* over two orders of magnitude, corresponding to a force quantification range of ≈21 pN (9‐30 pN). To further improve force quantification while minimizing phototoxicity, a brighter fluorogenic PAINT imager with *k_off_(F)* in the 0.1‐10 s range would be needed. While higher laser power could enhance spatial resolution, it induces rapid phototoxicity onset (within ≈5 min), potentially confounding mechanical readouts with biological damage. Thus, our qtPAINT approach prioritizes biological integrity over maximizing spatial and temporal resolution.

Photobleaching sets the lower bound for accurate *k_off_* determination in qtPAINT since slower unbinding events have a greater chance of being photobleached. To assess this, we measured the photobleaching rate of MB imagers bound to a 15 nt target strand (Figure S15, Supporting Information) under 2–10 mW illumination power. At 2 mW, the photobleaching rate (0.012 s^−1^) matches the *k_off_* of MB imagers at 9 pN (Figure 2b). At 10 mW, this rate increases to 0.039 s^−1^, corresponding to 13.8 pN, below the forces experienced in >99% of detected events. Hence, under the low‐power illumination used to minimize phototoxicity, photobleaching is negligible for the vast majority of tension events.

Like all DNA‐PAINT methods, qtPAINT requires time to accumulate sufficient localizations for super‐resolution imaging.^[^ ^44^ ^]^ While this is straightforward for fixed samples, it is a challenge for live cells where adhesive structures are dynamic and mobile. Tension PAINT does not record the start or end of mechanical events, but only reports the presence of a force high enough to unfold the probe at the time of imager binding. While qtPAINT cannot overcome this limitation, it leverages unbinding kinetics to extract force magnitude information that standard tension PAINT cannot provide.

Additional limitations arise from the statistical requirements of qtPAINT. A sufficient number of binding events must be collected to accurately calculate *k_off_*. This timescale must be balanced against the timescales of cellular force dynamics. Recent studies showed that the force loading rate in integrin adhesions can range from 0.5‐4 pN s^−1^, reaching >50 pN in 10–100 s.^[^ ^45^, ^46^, ^47^, ^48^ ^]^ Because qtPAINT cannot continuously track a single force event, we assume a constant force profile over each mechanical event trace, making it difficult to resolve force dynamics <10 s resolution. Moreover, tension PAINT only reports whether the DNA hairpin probe is unfolded at the time of imager binding, lacking information about the hairpin state between imager binding events.

The dynamic nature of the integrin‐RGD interaction, with receptor‐ligand unbinding occurring within 0.1‐20 s under varying forces^[^ ^49^, ^50^ ^]^, further complicates these measurements. Integrin clusters can sequentially engage the same DNA hairpin probe, producing a time‐averaged force signal at a given location rather than a record of single‐integrin dynamics. Low‐force events require longer observation times to accumulate enough imager‐binding statistics, potentially leading to an under‐representation of low‐force events that are short‐lived. While higher temporal resolution is challenging, further improvement is possible by adopting faster PAINT strategies to increase statistics over shorter periods. While loading rate studies^[^ ^45^, ^46^, ^47^, ^48^ ^]^ examined forces reaching discrete thresholds, near‐constant force events could be potentially overlooked. Currently, we have little knowledge of the population distribution of loading rate or different force dynamics. In this regard, qtPAINT excels at measuring steady or slow‐varying forces, whereas single‐molecule approaches are better suited for capturing fast‐force dynamics. Each technique thus offers a complementary perspective of the integrin dynamics.

Finally, averaging issues are common to all tension imaging methods. Without super‐resolution, high‐density events remain indistinguishable within a diffraction‐limited spot, resulting in force averaging. PAINT disperses spatial density over time, resulting in temporal averaging. This inherent interplay between spatial, force, and temporal resolution remains a fundamental challenge in tension imaging.

## 4 Conclusion

Compared to previous tension PAINT methods,^[^ ^30^, ^33^, ^34^ ^]^ qtPAINT introduces two additional dimensions of information: force magnitude and temporal dynamics. This allows molecular tension events to be mapped at ≈30 nm spatial resolution, a minute‐scaled temporal resolution, and a 9–30 pN force quantification range. This is achieved while maintaining low phototoxicity for hour‐long live‐cell observations. We demonstrate that qtPAINT can resolve spatio‐temporal‐magnitude details of molecular forces across diverse cellular states: polarized cells with mature focal adhesions, stationary cells with scattered adhesion, and motile cells undergoing directed migration. Notably, qtPAINT is able to capture single receptor‐ligand events of few pN force fluctuations at the leading and trailing edges of migrating cells, a detail unattainable with existing tension imaging techniques.

The primary contribution of this work is the concept that intrinsic DNA unbinding kinetics can reveal force information in tension imaging. While this study serves as a first implementation, we anticipate future advancements that enhance spatial and temporal resolutions. For example, brighter fluorophores and optimized imager kinetics could boost information throughput and reduce phototoxicity. Integrating qtPAINT with methods like MINFLUX^[^ ^51^ ^]^ may further improve spatial and temporal resolution, especially in regions of high mechanical event density. Finally, combining qtPAINT with biosensors for intracellular force and biochemical signaling will open new avenues for quantitative mechanotransduction research.

## Conflict of Interest

The authors declare no conflict of interest.

## Supporting information



Supporting Information

Supplemental Movie 1

Supplemental Movie 2

## Data Availability

The data that support the findings of this study are available from the corresponding author upon reasonable request.
