# Supplementary material for: Quantitative Super‐Resolution Imaging of Molecular Tension
Source: Adv Sci (Weinh). 2025 Apr 17;12(28):2408280. doi: 10.1002/advs.202408280 (PMC12302535; doi:10.1002/advs.202408280)
Supplement: Supplementary file 1 — Supporting Information [file ADVS-12-2408280-s002.pdf]

ADVANCED SCIENCE Open Access

Supporting Information

for *Adv. Sci.*, DOI 10.1002/advs.202408280

Quantitative Super-Resolution Imaging of Molecular Tension

*Seong Ho Kim, Adam B. Yasunaga, Hongyuan Zhang, Kevin D. Whitley and Isaac T. S. Li**

Supporting Information

Quantitative super-resolution imaging of molecular tension

Seong Ho Kim[1,2], Adam B. Yasunaga[1], Hongyuan Zhang[1], Kevin D. Whitley[3], Isaac T.S. Li[1]*

[1] Department of Chemistry, The University of British Columbia, Kelowna, BC, V1V 1V7, Canada

[2] Department of Chemistry and Advanced Materials, Gangneung-Wonju National University, Gangneung 25457, Republic of Korea

[3] Centre for Bacterial Cell Biology, Biosciences Institute, Newcastle University, Newcastle upon Tyne, NE1 7RU, UK

* corresponding author: isaac.li@ubc.ca

Contents **Page**

Materials and Method 2

Figure S1. Colocalization of Paxillin with tension. 4

Figure S2. Colocalization of Talin-1 with tension. 5

Figure S3. RGD-mediated cell adhesion and sensor-mediated tension generation 6

Figure S4. Spatial resolution of qtPAINT. 7

Figure S5. Analysis of subcellular structural mechanics in a polarized cell. 8

Figure S6. Sample traces of qtPAINT events. 9

Figure S7. Statistics of qtPAINT traces in the edge and center regions and force uncertainty. 10

Figure S8. Analysis of subcellular mechanics in an unpolarized cell. 11

Figure S9. Quantitative tension mapping of stationary HEK293 cells. 12

Figure S10. Quantitative tension mapping of stationary MDA-MB-231 cells. 13

Figure S11. Analysis of tension distribution across multiple cells 14

Figure S12. Effect of blebbistatin on tension changes. 15

Figure S13. Unbinding kinetics remain unchanged in fixed cells over time. 16

Figure S14. Overview of background filtering process. 17

Figure S15. Photobleaching analysis of Cy3B-BHQ2 MB imager. 18

Table S1. DNA sequences and modifications. 19

Table S2. qtPAINT imaging parameters. 20

References 21

Materials and Method

Materials

DNA: DNA oligonucleotides were ordered from Integrated DNA Technologies and Ella Biotech with HPLC purification. Their sequences are listed in Table S1.

Chemicals and reagents: NeutrAvidin (31000) and glass coverslip (60 x 24mm, 12-548-5P) were purchased from Fisher Scientific. PEG (mPEG-SVA, 5k) and biotin-PEG (biotin-PEG-SVA, 5k) were acquired from Laysan Bio. Tris(2-carboxyethyl)phosphine hydrochloride (c4706) was purchased from Sigma-Aldrich. N-(3-(Trimethoxysilyl)propyl)ethylenediamine (1760-24-3) and sodium bicarbonate (470302-444) were acquired from VWR international. BSA (9048-46-8) was obtained from Tocris Bioscience. Sulfo-SMCC (sulfosuccinimidyl 4-(N-maleimidomethyl) cyclohexane-1-carboxylate) (786-082) was acquired from G-Biosciences. Micro Bio-Spin P-6 Gel Columns (7326200) were ordered from Bio-Rad. KOH (BDH9262), MeOH (BDH1135), Acetic acid (BDH3094), NaCl (BDH9286), $MgCl_2$ (BDH9244), $CaCl_2$ (BDH9224), Tris (BDH7729) and HEPES (0511) were all purchased from VWR International. EDTA (46-034-CI) was ordered from Corning Life Sciences.

Peptide: Cyclo[Arg-Gly-Asp-D-Phe-Lys(PEG-PEG)] (PCI-3696-PI) was purchased from vivitide.

Imaging supplies: Culture-Inserts 4 Well for self-insertion (80489) was ordered from ibidi. Double-sided tape (3M237) was obtained from 3M Scotch.

Buffers and media: HBSS (04-315Q) was purchased from Lonza. TM5 buffer (50 mM NaCl, 5 mM $MgCl_2$, 10 mM Tris-HCl pH 8.0) was used for DNA preparation. HHMC buffer (1x HBSS, 0.1 mM $MgCl_2$, 0.1 mM $CaCl_2$, 10 mM HEPES pH 7.4) and HHM5 buffer (1x HBSS, 5 mM $MgCl_2$, 10 mM HEPES pH 7.4) were used for imaging and surface washing.

Cell Culture Supplies: Ham's F-12K Medium (21127022) and Penicillin/Streptomycin (SV30010) were purchased from Thermo Fisher Scientific. DMEM (12-604F) was purchased from Lonza Bioscience. FBS (97068-085) was ordered from VWR international. Lipofectamine 3000 (L3000015) was purchased from Invitrogen. Blebbistatin (B0560-1MG) was ordered from Sigma-Aldrich.

Bioconjugation of cRGDfk to thiol-modified DNA

cRGDfk was conjugated to the ligand DNA through the sulfo-SMCC crosslinker. 11.2 mM cRGDfk (50 µL) in PBS was reacted with 23 mM sulfo-SMCC (10 µL) in ultra-pure water at room temperature for 2 hrs, creating a maleimide-modified cRGDfk peptide. Simultaneously, 1 mM of thiol-modified DNA (20 µL) in ultra-pure water was reduced using TCEP (5 µL, 1x PBS, 20 mM TCEP, and 20 mM EDTA). Following reduction, the DNA was purified using a gel filtration column (Micro bio-spin P-6 columns, Bio-Rad, USA) to remove unreacted reagents. The maleimide-modified cRGDfk was then reacted with the reduced DNA at 11.5:1 molar ratio of cRGDfk:DNA at 4 °C overnight. The excess cRGDfk was removed by another round of gel filtration. The DNA hairpin sensor was assembled by mixing it with the hairpin, anchor, and RGD-ligand strand at a 1.1:1:1 molar ratio in the TM5 buffer for 1 hr at room temperature.

Cell culture and transfection

Culture media were supplemented with 1% penicillin/streptomycin and 10% fetal bovine serum. CHO-K1 cells were cultured in F-12K medium. HEK293 and MDA-MB-231 cells were cultured in DMEM. Cells were maintained at 37 °C and 5% CO_2 and passaged every 2-3 days. Paxillin (EGFP-m-Paxillin, Addgene #80023) and Talin1 (GFP-Talin1, Addgene #26724) plasmids were transfected with lipofectamine 3000 according to the manufacturer's instructions. Cells were incubated for 40 hrs after transfection.

Surface preparation for tension imaging

The imaging surface was prepared according to previously published protocols[1]. Briefly, glass coverslips were cleaned using KOH and methanol. Air plasma (0.4 mTorr for 70 s) was treated to the surface to render it hydrophilic. For amino-silanization, the coverslips were incubated in 94 mL of methanol, 5 mL of acetic acid, and 1 mL of aminosilane for 1 hr in the dark. The coverslips were rinsed three times with methanol

and water. After drying coverslips using nitrogen gas, mPEG-succinimidyl valerate (SVA) and biotin-PEG-SVA (20:1 molar ratio) in sodium bicarbonate buffer were treated to the surface and incubated in the moisture chamber (overnight). Coverslips were rinsed with ultra-pure water and dried under nitrogen gas. A 4-well insert was attached to the coverslip and mounted with a 3D-printed adapter for imaging. The surface was passivated with 1% BSA and washed with imaging buffer. For the immobilization of the hairpin tension probe, 100 µg/mL of neutravidin was incubated on the surface for 10 min and washed with HHMC buffer. 100 nM hairpin tension probes were incubated on the neutravidin-functionalized surface for 20 min and washed with HHMC buffer. Cells at 2×10^5 cells/mL density were seeded onto the hairpin-probe modified surface and allowed to spread for 1 hr at 37 °C and 5% CO_2. Following washing with HHMC buffer, MB imagers were added and imaged under the conditions detailed in Table S2.

Super-resolution tension PAINT imaging

Tension imaging was performed on Olympus IX83 inverted microscope with a custom-built TIRF system. For the excitation of MB imagers (Cy3B fluorophore and BHQ2 quencher), a 532 nm laser (Spectra-Physics, Excelsior 532) was employed. PAINT movies were captured using an Andor iXon Ultra 897 EMCCD camera combined with a 100x oil-immersion TIRF objective (Olympus, UPLAPO100XOHR NA 1.49). A pixel size of 160 nm was calibrated using an NBS 1963A resolution test target (Thorlabs R2L2S1P). All imaging experiments were performed at room temperature. Individual tension events were localized using the UNLOC algorithm as an ImageJ plugin[2] and tracked using custom Matlab code with a detection radius of 160 nm. Tracks with a single localization event are removed from subsequent analysis. Sparse non-specific events outside of the cell area are removed by cluster filtering from subsequent grid analysis. The force-dependent hybridization kinetic model was based on a previously published and experimentally validated model. [3] For photobleaching measurement, 15 nt linear imagers (100 nM) was immobilized on the surface. Cy3B-MB (1 nM) was excited on the surface using a 532 nm laser at 10 mW power. Imaging was conducted in HHM5 buffer.

Cells exhibit nuclease activity that can vary depending on the cell line, potentially interfering with experiments. To minimize this effect, we conducted our experiments within 1 hour of initial cell seeding, similar to established protocols from other DNA-based molecular force probe studies.[4,5]

Confocal imaging

Transfected cells were cultured on the hairpin-probe-functionalized surface and incubated for 2 hrs at 37 °C in a 5% CO_2 environment. Cell imaging was conducted using a confocal microscope (Olympus, FV3000) using a UPLXAPO 60X objective (NA 1.42). The imaging involved dual excitation wavelengths: 561 nm for tension and 488 nm for GFP-labelled FA proteins. Time-lapse imaging capturing both tension and Paxillin dynamics was performed over 25 minutes, with images taken at 10-second intervals.

Statistics and reproducibility

Independent imaging sessions for stationary and motile CHO-K1 cells and stationery HEK293 and MDA-MB-231 cells were conducted at least three times. Statistical significance was determined using a one-way ANOVA. The presented data are representative of results obtained from at least three replicates. In Figures 2n, 3f, 3m, S5e, S9d, S10d, and S11e, red lines in the box plot represent the median value, the top and bottom of the boxes represent 75th and 25th percentiles of the sample, respectively, and the ends of the whiskers indicate the max and min values excluding outliers.

For force quantifications, traces with less than 5 binding events were removed to ensure force quantification uncertainty do not exceed ±2 pN. See Figure S7f, g for more details.

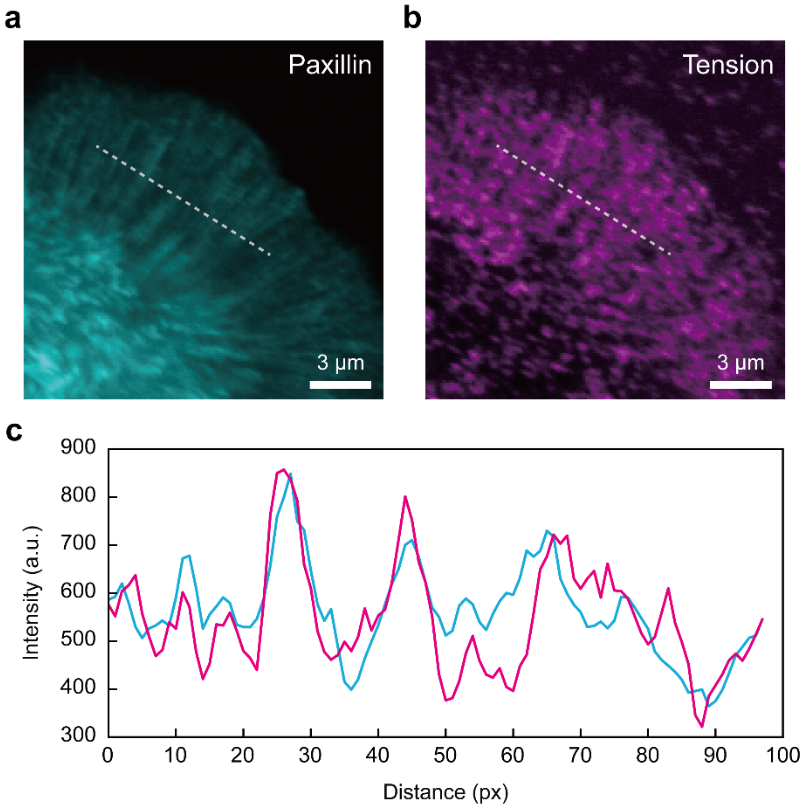

Figure S1. Colocalization of paxillin with tension. **(a, b)** Averaged images of paxillin (a) and tension (b) in CHO-K1 cells overexpressing Paxillin-GFP. Tension was imaged using 150 nM MB imager under confocal microscopy. **(c)** Line scans of images showing Paxillin (cyan) and tension (magenta). The fluorescence intensity along the dotted line in (a, b) was quantified using ImageJ, showing correlation between the presence of paxillin and force production.

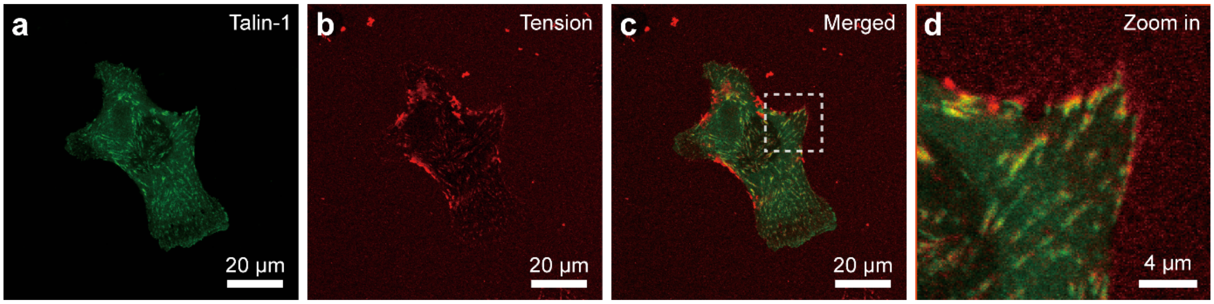

Figure S2. Colocalization of Talin-1 with tension. Confocal images of **(a)** Talin-1-GFP and **(b)** tension in CHO-K1 cells overexpressing Talin-1-GFP. Tension was imaged using 150 nM MB imager. **(c)** Merged images from (a) and (b), **(d)** Zoom in image of the boxed region in (c).

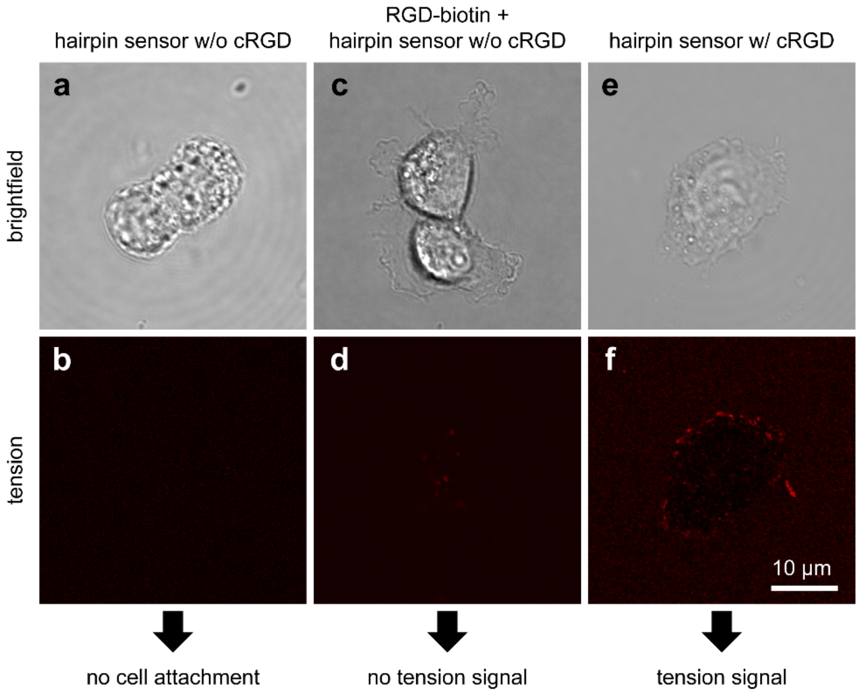

Figure S3. RGD-mediated cell adhesion and sensor-mediated tension generation. Representative brightfield and tension images of cells on surfaces with DNA hairpin probe without RGD **(a, b)**, with RGD-biotin **(c, d)**, and DNA hairpin probe with RGD (**e, f**), all in the presence of 100 nM MB imager.

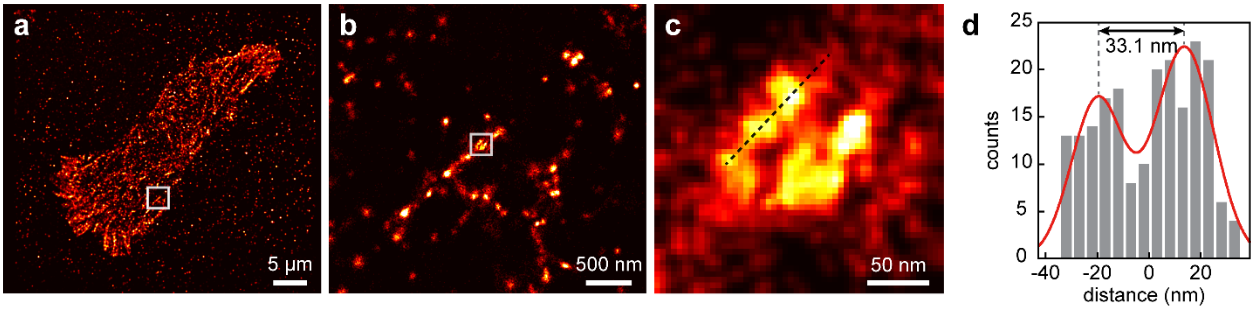

Figure S4. Spatial resolution of qtPAINT. **(a-c)** Super-resolution tension images, featuring progressively magnified views of boxed regions (white solid box). **(d)** The cross-sectional profile along the black dashed line in (c), being able to resolve features with a peak-to-peak distance of 33.1 nm.

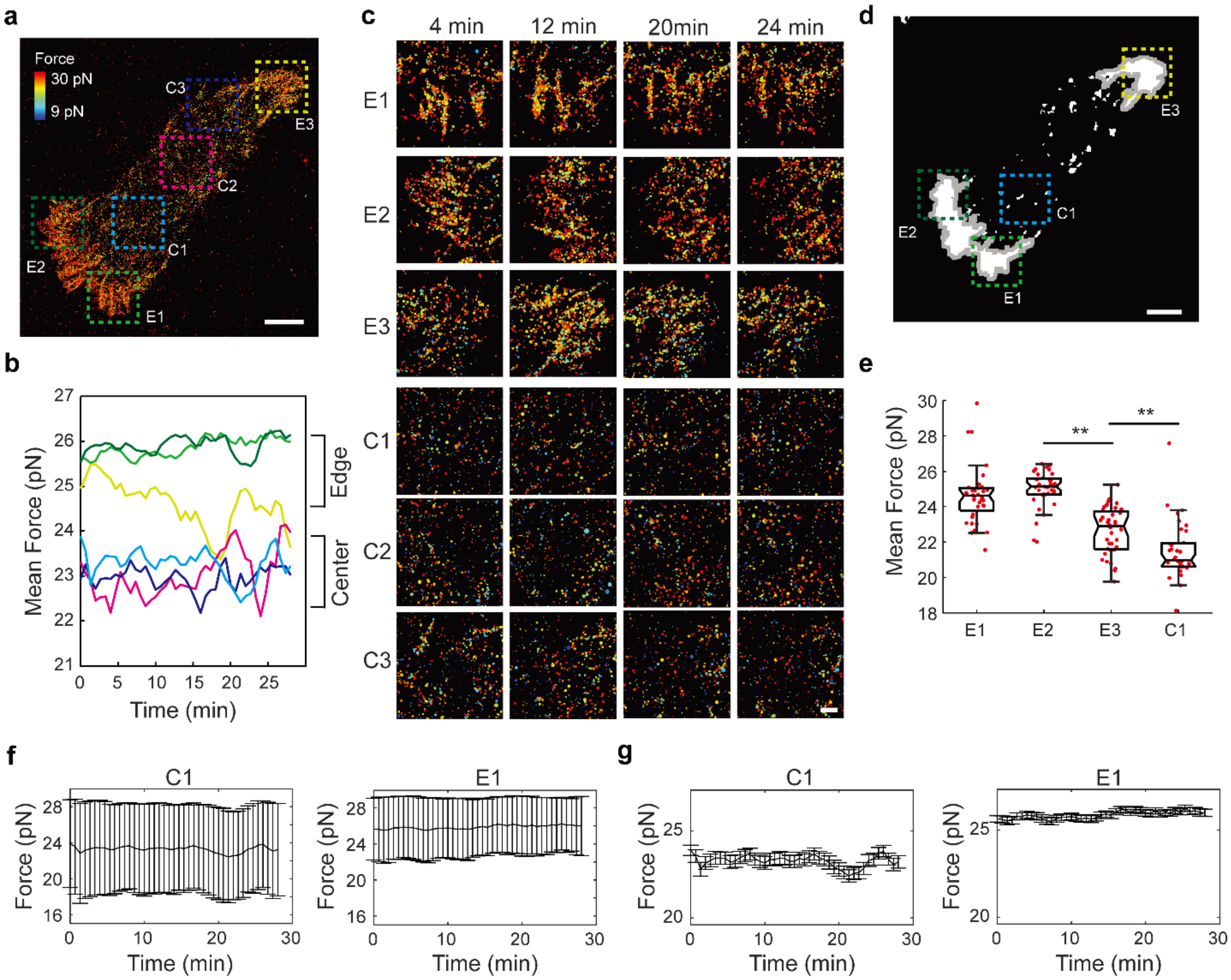

Figure S5. Analysis of subcellular structural mechanics in a polarized cell. **(a)** Quantitative tension map with three boxed regions of interest in the edge (E1, E2, E3) and center (C1, C2, C3). **(b)** Mean force trajectories of each boxed region from the edge and center in (a). **(c)** Time series of zoomed-in views of the edge and center from each boxed region in (a). **(d)** A mature FA masked image with three boxed regions in the edge and one boxed region in the center. **(e)** Comparison of mean force values in (d). ** indicates $p<0.01$. **(f)** Mean force values with error bars indicating standard deviation of all force events within C1 and E1 regions, to indicate the spread of force events. **(g)** Mean force values with error bars indicating the standard error of the mean in C1 and E1 regions, to indicate statistically significant overall force fluctuation in the region. Scale bars: 5 μm (a, d) and 1 μm (c).

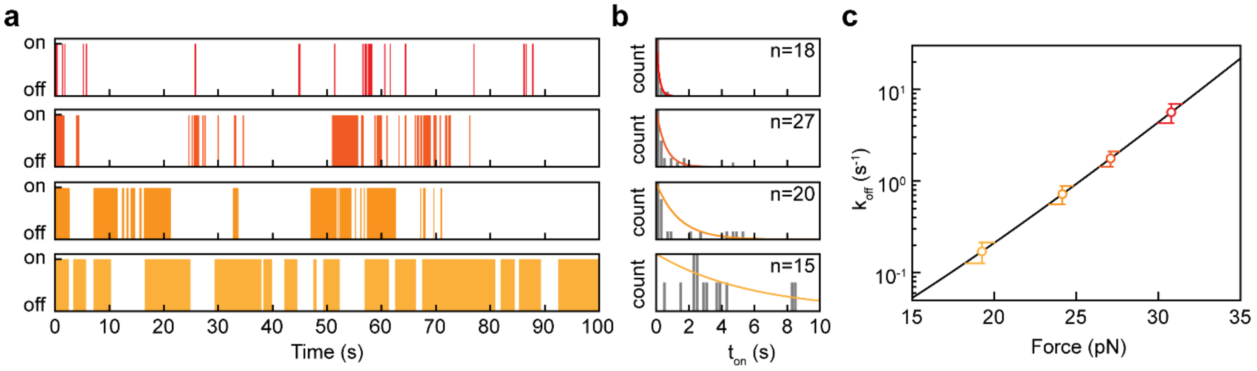

Figure S6. Sample traces of PAINT events. **(a)** Four long traces showing different levels of k_{off} from fast (top) to slow (bottom). Traces only show the first 100 s for comparison. Note that the events can be the result of multiple engagement and dissociation of integrin acting on the same hairpin probe over time, as PAINT cannot distinguish hairpin state between imager binding events. **(b)** The binding time (t_{on}) histogram for each trace in (a). The number of events per trace is indicated by n. The distribution is assumed to follow single-exponential decay (solid lines) with a decay constant (τ) calculated by the mean of t_{on}, as $\tau = \langle t_{on} \rangle$ for distributions following exponential decay. **(c)** Mapping of the four k_{off} from (a) onto the force calibration curve model (black solid line). The error bars in k_{off} is estimated as the standard error of the mean, where they intersect with the solid line provides an estimation in the uncertainties in force they report.

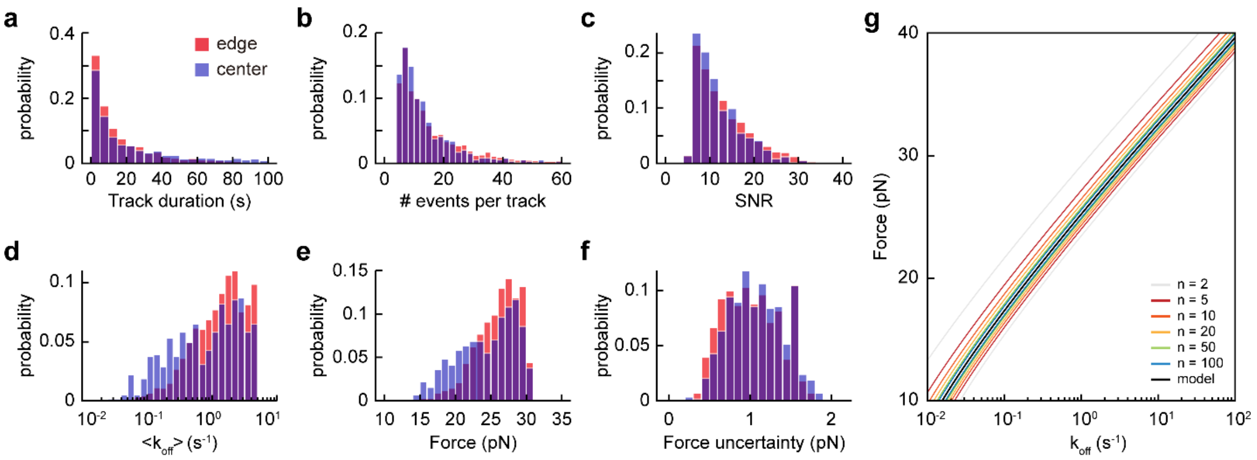

Figure S7. Statistics of qtPAINT traces in the edge and center regions and force uncertainty. (Figure 2e-j, ROIs 1 and 2) **(a)** Histogram of the durations of traces from the edge and center. **(b)** Histogram of the number of event per trace, with a minimal cutoff at 5 events per trace. **(c)** The signal-to-background ratio of individual binding events showing similar distribution between the two regions. **(d)** Distribution of $<k_{off}>$ values at the center and the edge, where higher k_{off} indicate greater tension (Figure 2i, j). **(e)** Distribution of force derived from (d). **(f)** Distribution of force uncertainty based on the number events per trace. All events are within 2 pN uncertainty of the reported value. **(g)** Systematic investigation of how the number of events (from n = 2 to 100) affect the uncertainty in the reported force values.

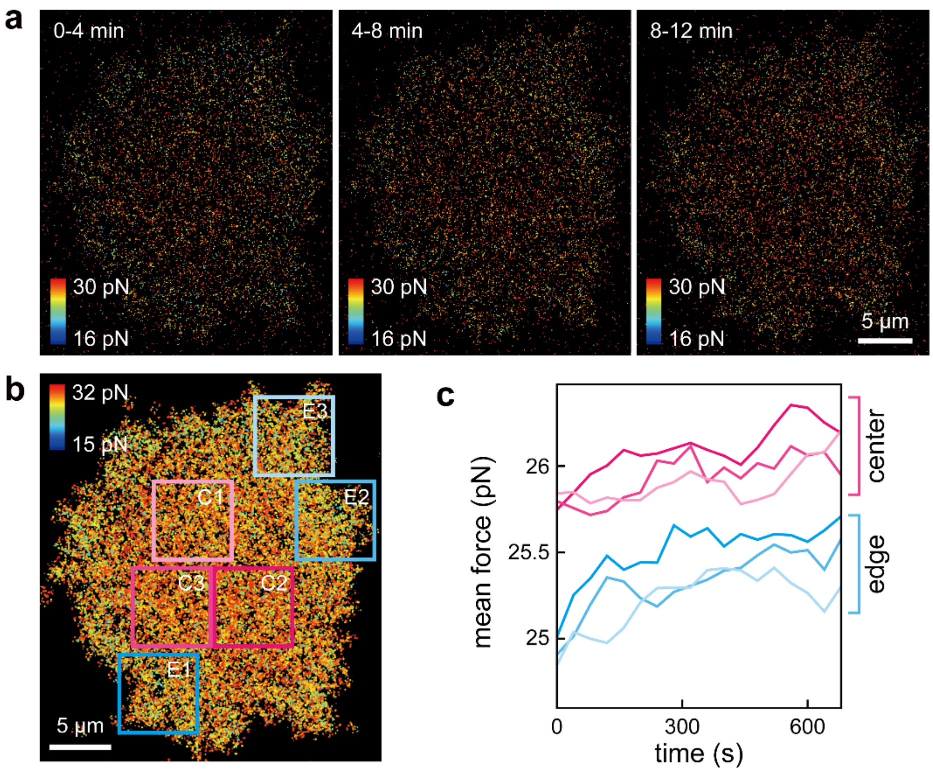

Figure S8. Analysis of subcellular mechanics in an unpolarized cell. **(a)** The distribution of force within a stationary CHO-K1 cell as time progresses. The intervals of 0, 4, and 8 min correspond to the time periods over which force integration was conducted, covering the ranges of 0-4 min, 4-8 min, and 8-12 min, respectively. **(b)** Quantitative tension map with three boxed regions of interests in the edge (E1, E2, E3) and center (C1, C2, C3). **(c)** Mean force trajectories of each boxed region from the edge and center in (b).

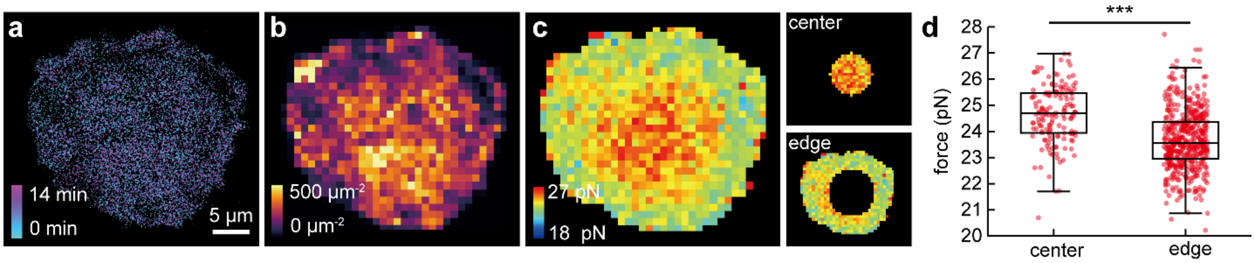

Figure S9. Quantitative tension mapping of stationary HEK293 cells over 14 min. The resulting qtPAINT results were presented in **(a)** time, **(b)** event density grid, and **(c)** force grid maps, with color bars denoting the respective range. The center and edge were selected visually to demonstrate the force in the central region is greater than the rest. **(d)** Quantification of the force value in each grid from the center and edge regions, with statistical significance (*** = $p < 0.001$).

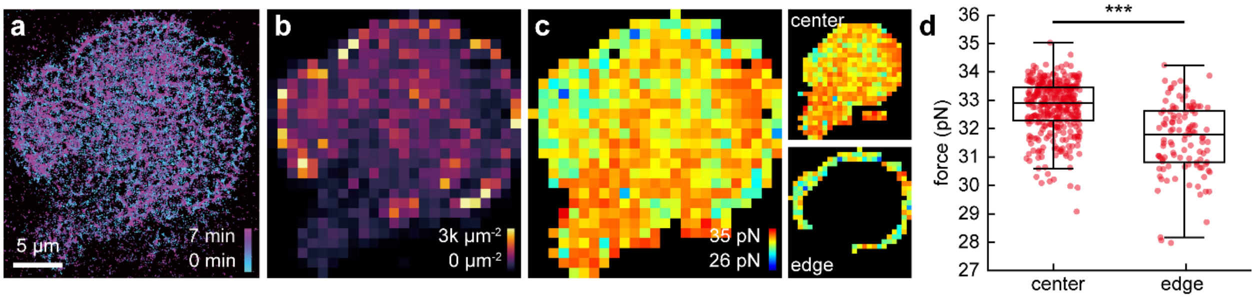

Figure S10. Quantitative tension mapping of stationary MDA-MB-231 cells over 7 min. The resulting qtPAINT results were presented in (a) time, (b) event density grid, and (c) force grid maps, with color bars denoting the respective range. The center and edge were selected visually to demonstrate the force in the central region is greater than the rest. (d) Quantification of the force value in each grid from the center and edge regions, with statistical significance (*** = $p < 0.001$).

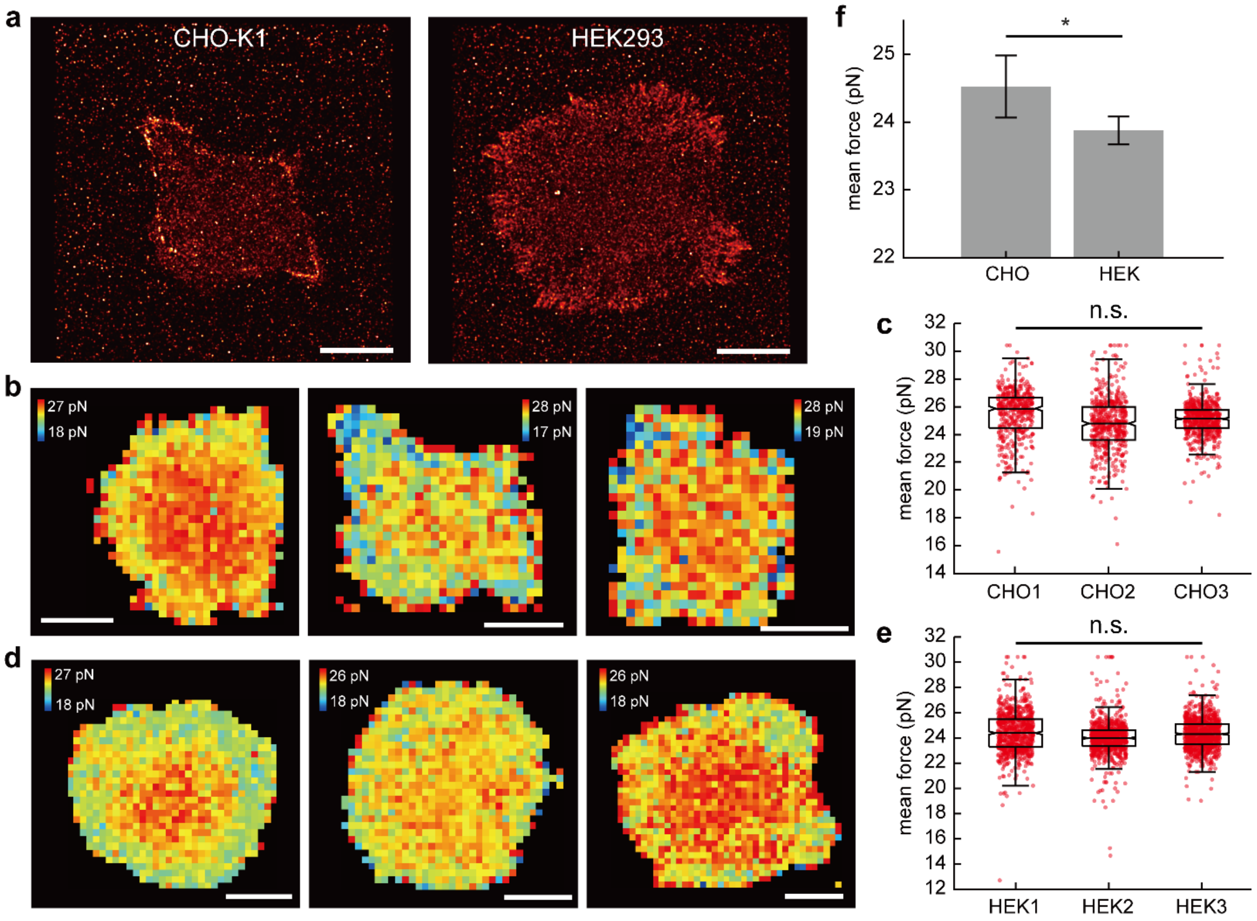

Figure S11. Analysis of tension distribution across multiple cells. **(a)** Representative super-resolution images of CHO-K1 and HEK293 cells. **(b)** Coarse-grained grid maps showing mean force in 3 different CHO-K1 cells. **(c)** Comparison of mean force values in the three CHO-K1 cells in (b). **(d)** Coarse-grained grid maps showing mean force in 3 different HEK293 cells. **(e)** Comparison of mean force values in the three HEK293 cells in (d). **(f)** Comparison of average mean force values in CHO-K1 and HEK293 cells. * indicate $p<0.05$. Scale bars: 10 μm (a, b, d).

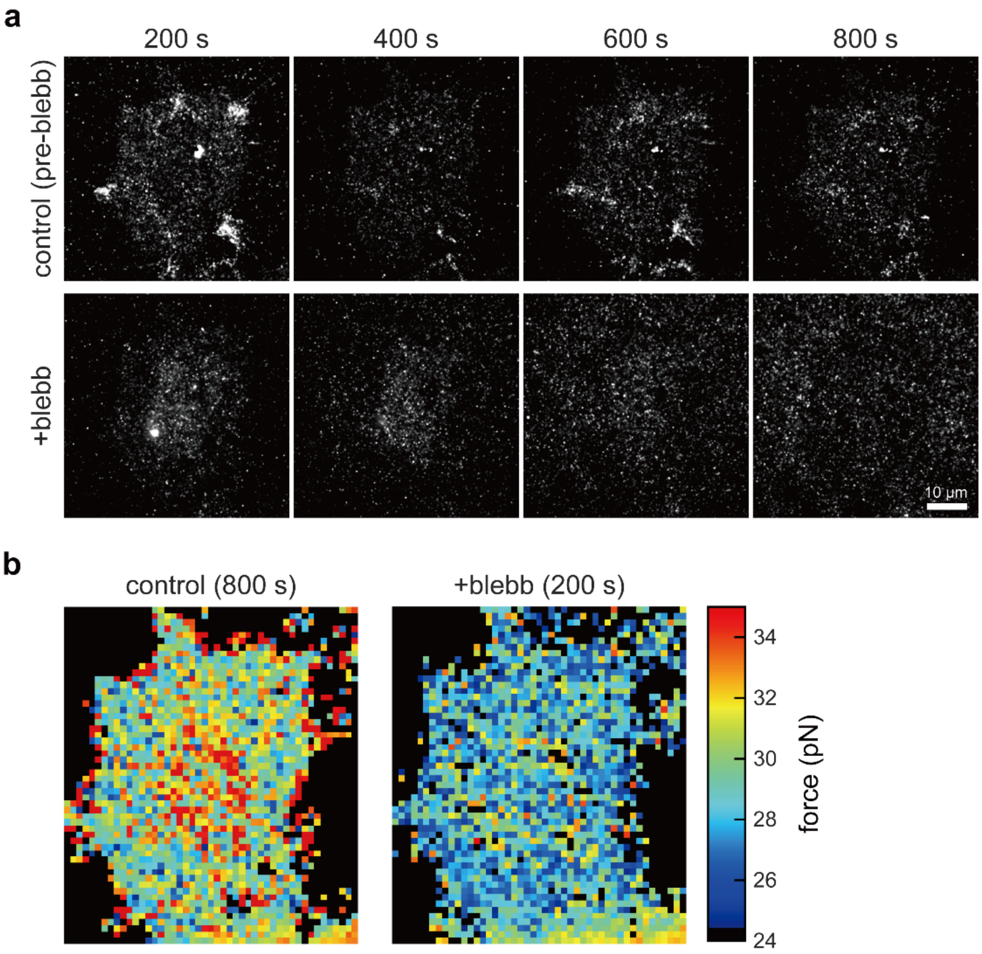

Figure S12. Effect of blebbistatin on tension changes. **(a)** Tension images acquired before and after the incubation of blebbistatin (25 μM). Control HEK293 cells were initially imaged using qtPAINT for 800 s. Subsequently, blebbistatin was added, and the cells were incubated for 5 min before imaging the drug-treated cells. Images were averaged at 200 s intervals. **(b)** Force maps before and after the incubation of blebbistatin. Force maps were generated from control cell acquired for 600~800 s and drug-treated cell acquired for 0~200 s. Scale bars: 10 μm (a) and 5 μm (b).

a
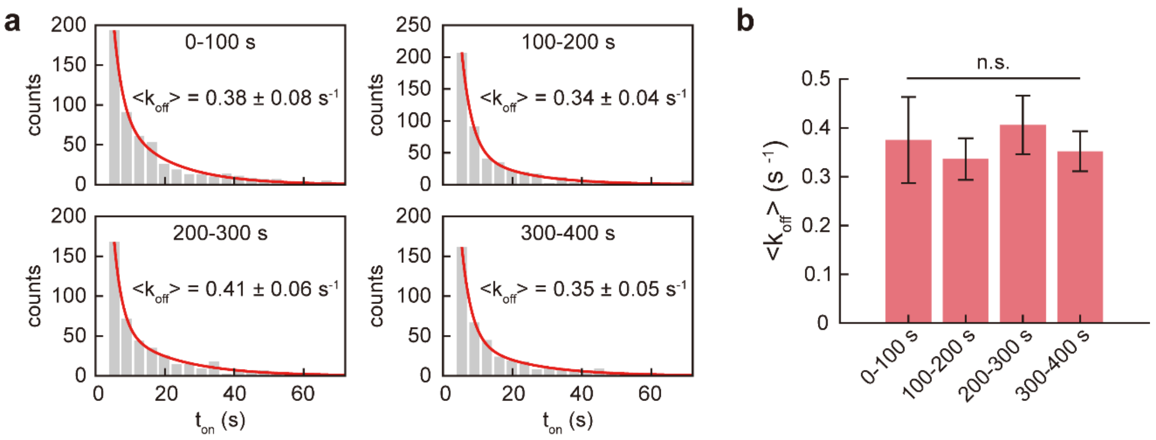

Figure S13. Unbinding kinetics remain unchanged in fixed cells over time. **(a)** Histograms of t_{on} of mechanical events under the fixed CHO-K1 cell with 100-second intervals. Single exponential fits (red solid lines) to extract the average k_{off} for each time period. The error in k_{off} values indicate 95% confidence interval of the exponential fit. **(b)** No statistical significance were found between the mean k_{off} values across the time periods.

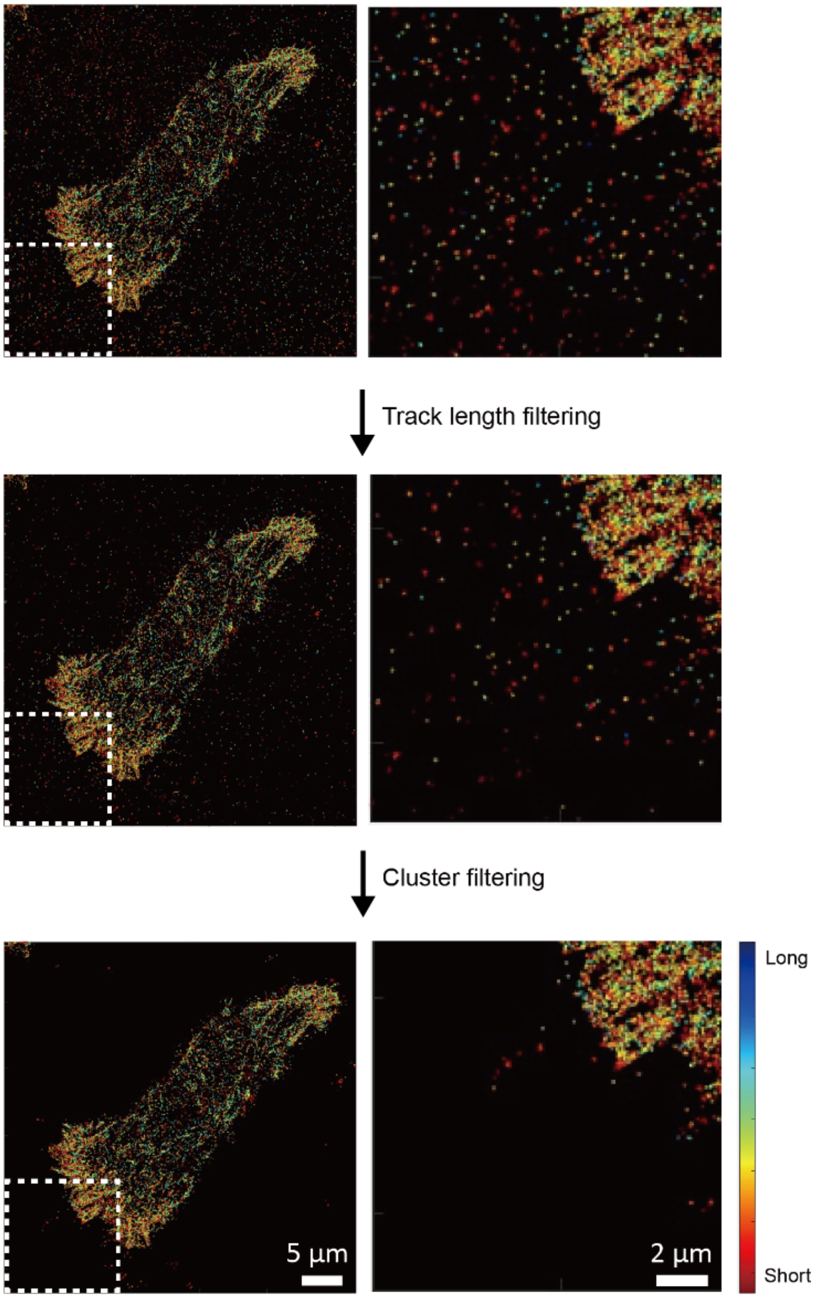

Figure S14. Overview of background filtering process. Non-mechanical signals were eliminated using track length and cluster filtering. Tracks with short length (< 5 events) and insufficient events for clustering were excluded from the analysis of mechanical events, ensuring that non-mechanical signals did not impact the actual tension signal beneath the cells. The color bar represents track length.

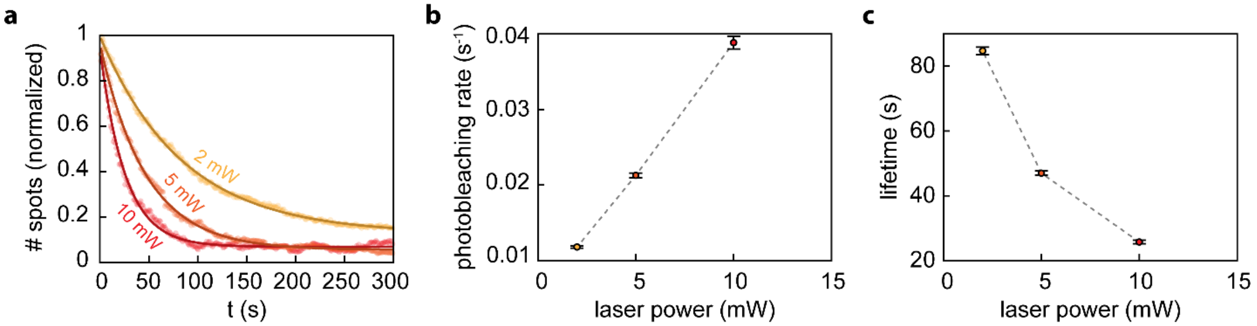

Figure S15. Photobleaching analysis of Cy3B-BHQ2 MB imager. Imagers are bound to long target strands (15 nt linear target, Table S1) to facilitate long binding lifetime in order to understand the photobleaching rate. **(a)** Normalized number of fluorophores remaining on surface over time under 2 mW (yellow, n=868), 5 mW (orange, n=725), and 10 mW (red, n=329) illumination powers (used in the current study). Each decay curve is fitted by a single exponential decay function to obtain **(b)** the photobleaching rate (0.012±0.002 s^{-1}, 0.021±0.003 s^{-1}, 0.039±0.008 s^{-1}), and **(c)** mean lifetimes (85±1 s, 47.0±0.7 s, and 25.7±0.6 s). Error bars indicate 95% confidence interval of either the photobleaching rate or the lifetime values from fitting in (a). The photobleaching rate is about an order of magnitude lower compared to the k_{off} of the imagers, demonstrating the kinetics observed in live cell environment is a result of unbinding and not photobleaching, as indicated shown in Figure 2b.

Table S1. DNA sequences and modifications.

Name	Sequences (5' - 3')	Modification	Vendors
MB imager	GTA CGC GCC AAA AAA AGG TAC	5'-Cy3B<br>3'-BHQ2	Ella Biotech
Ligand	TTT GCT GGG CTA CGT TCG GCT CTT	5'-5ThiolMC6-D	IDT
Anchor	CGC ATC TGT GCG GTA TTT CAC TTT	3'-biotin	IDT
P9 hairpin	GTG AAA TAC CGC ACA GAT GCG TTT GTA CGC GCC AAA AAA AGG CGC GTA C TTT AAG AGC CGA ACG TAG CCC AGC	none	IDT
15 nt linear target	GTG AAA TAC CGC ACA GAT GCG TTT GCG GAC AAC ATT TTT TGG CGC GTA C TTT AAG AGC CGA ACG TAG CCC AGC	none	IDT

Note: 5ThiolMC6-D refers to thiol modified 5' DNA via a 6-C linker. The MB binding site is underlined in P9 hairpin and 15 nt linear targets. The anchor, ligand and P9 hairpin strands assemble to the full hairpin sensor, where the anchor strand containing biotin is for surface immobilization and ligand strand further modified with the integrin cRGDfk ligand (See Figure 1a).

Table S2. qtPAINT imaging parameters.

Data in images	Parameters	Laser power (532 nm)
Fig. 2d-f,<br>Fig. S4, S5	5 fps, 10000 frames, 0.1 mM $MgCl_2$, 0.1 mM $CaCl_2$,<br>25 nM MB imager, 100 nM Hairpin sensor P9	2 mW
Fig. 3g-k	5 fps, 20000 frames, 5 mM $MgCl_2$,<br>20 nM MB imager, 100 nM Hairpin sensor P9	2 mW
Fig. 3a-e,<br>Fig. S8, S11b	50 fps, 40000 frames, 5 mM $MgCl_2$,<br>100 nM MB imager, 100 nM Hairpin sensor P9	10 mW
Fig. 3n, o,<br>Fig. S12, S11d	50 fps, 40000 frames, 5 mM $MgCl_2$,<br>100 nM MB imager, 100 nM Hairpin sensor P9	5 mW
Fig. S10	20 fps, 8000 frames, 5 mM $MgCl_2$,<br>200 nM MB imager, 100 nM Hairpin sensor P10	5 mW
Fig. S13	10 fps, 4500 frames, 0.1 mM $MgCl_2$, 0.1 mM $CaCl_2$,<br>100 nM MB imager, 100 nM Hairpin sensor P9	5 mW

References

[1] S. H. Kim, I. T. S. Li, *Angew Chem Int Ed* **2023**, *62*, e202217028.
[2] S. Mailfert, J. Touvier, L. Benyoussef, R. Fabre, A. Rabaoui, M.-C. Blache, Y. Hamon, S. Brustlein, S. Monneret, D. Marguet, N. Bertaux, *Biophys J* **2018**, *115*, 565–576.
[3] K. D. Whitley, M. J. Comstock, Y. R. Chemla, *Nucleic Acids Research* **2017**, *45*, 547–555.
[4] X. Wang, T. Ha, *Science* **2013**, *340*, 991–994.
[5] Y. Zhang, C. Ge, C. Zhu, K. Salaita, *Nat Commun* **2014**, *5*, 5167.
